# Features of Elite Athletes' High and Asymmetric Quadriceps Angles: A Cross‐Sectional Study

**DOI:** 10.1002/hsr2.70909

**Published:** 2025-06-11

**Authors:** Kohei Hikawa, Reia Shimizu, Saeko Takahashi

**Affiliations:** ^1^ Department of Sports Medicine Japan Institute of Sports Sciences Tokyo Japan

**Keywords:** elite athlete, injury prevention, knee, quadriceps angle

## Abstract

**Objective:**

To conduct a large‐scale survey of Quadriceps angles (Q‐angles) in elite athletes and clarify the characteristics and potential implications of high and asymmetric Q‐angles for injury risk.

**Design:**

This cross‐sectional study utilized Q‐angle data obtained during medical check‐ups of elite national team male and female athletes at our facility over a 20‐year period.

**Methods:**

We analyzed the height and asymmetry of the Q‐angle in 11,616 elite athletes, examining sex differences through logistic regression analysis.

**Results:**

Analysis of 23,094 knees revealed a significant prevalence of high Q‐angles in female athletes (13.2%) compared to male athletes (4.2%) with an odds ratio (OR) of 3.47 (*p* < 0.05, 95% confidence interval (CI): 3.13–3.84), and a higher rate of Q‐angle asymmetry in females (27.9%) than in males (22.7%) with an OR of 1.26 (*p* < 0.05, 95% CI: 1.16–1.34).

**Conclusion:**

Our findings highlight a significant gender disparity in high and asymmetric Q‐angles among elite athletes, underscoring the need for gender‐specific approaches in injury prevention and further research into the biomechanical effects of Q‐angle variances.

## 1 Introduction

The Quadriceps angle (Q‐angle) is the angle between the line from the anterior superior iliac spine to the center of the patella and the line from the center of the patella to the center of the tibial tuberosity; it is an evaluation index of static knee alignment, which is measured conveniently [1]. The Q‐angle is larger in female than in male among non‐athletes [2]. The Q‐angle differs between sexes. Further, there are differences between the Q‐angle in athletes of different sports [3, 4, 5] owing to the influence of the sports' characteristics. Fatahi et al. [3] and Shambaugh et al. [6] examined volleyball and basketball athletes, respectively, and found that an increase in the Q‐angle is associated with the occurrence of knee injuries. It is believed that if the Q‐angle exceeds the limit of 15°–20°, the knee extension mechanism becomes impaired, making the patella more prone to lateral displacement, leading to knee injuries such as patellofemoral pain [7, 8]. Additionally, Padasala et al. [9] compared runners with or without a history of anterior knee pain and found that the asymmetric Q‐angle between the left and right sides was associated with the presence or absence of knee injury. Furthermore, Moltubakk [10] and Vargas et al. [11] reported that asymmetry in the left and right knee morphology increases injury risks owing to an imbalance in muscle strength exertion. The Q‐angle has been considered to be a highly useful indicator for preventing knee injuries in athletes. In addition, focusing on the Q‐angle values and the presence or absence of a high Q‐angle and left‐right asymmetry facilitates a more practical understanding of the physical characteristics of the participant, which is crucial for preventing injuries.

Given this background, understanding the characteristics of athletes' Q‐angle is important both clinically and academically when considering knee injury prevention.　However, studies have mostly investigated the Q‐angle values in limited sports and at competitive levels and their relationship with knee injuries [3, 6]. Comprehensive research on elite athletes across various sports is limited. Elite athletes are exposed to greater mechanical stress on their knees due to their high performance, and are reported to suffer from knee injuries than athletes at other competitive levels [12, 13]. It has also been proposed that high levels of physical activity, accompanied by chronic repetitive stress and joint overuse, are likely the primary mechanisms for future knee osteoarthritis [14]. In other words, it is possible that knee alignment, such as the Q‐angle, may differ from general standards due to the sport‐specific movements and training that elite athletes perform on a daily basis. Thus, elite athletes need more information than athletes at other competitive levels. However, there is a lack of comprehensive research on elite athletes across different sports. Therefore, we believe that it is necessary to conduct comprehensive research on elite athletes. Hence, this study aimed to conduct a large‐scale investigation of the quadriceps angle (Q angle) in elite athletes and to determine the characteristics of high and asymmetric Q angles and their potential impact on injury risk. Reportedly, Quadriceps muscle mass is negatively correlated with Q‐angle [11]; hence, we hypothesized that elite athletes with certain physical characteristics may have a different alignment, such that they have a lower prevalence of high Q angles compared to non‐athletes and general athletes, as reported in previous studies. Additionally, female individuals are generally thought to have less muscle mass than male individuals, therefore, it is assumed that the positive rate of high Q‐angle is higher in female individuals than in male individuals. Similarly, a study has shown that the competitive characteristics of sports affect the Q‐angle [5, 15]. Consequently, we expected that asymmetry may be more prevalent in elite athletes with higher competitive ability.

## 2 Methods

### 2.1 Study Design

This cross‐sectional study investigated the data from medical check‐ups conducted at our institution for Japanese national athletes who competed in International competitions. Given that elite athletes often have different physical characteristics even within the same sport, depending on their individual sports style and position, we conducted a cross‐sectional study involving a broad cohort. This approach aimed to identify generalized patterns, including elevated and asymmetric Q‐angles among elite athletes. This medical check‐up is conducted to investigate the injury status and conditioning of athletes before participating in a competition, and includes a doctor's examination and alignment measurement by a physical therapist and an athletic trainer. Since medical check‐ups at our facility were mandatory before participating in international competitions, all athletes who participated in international competitions during the period were eligible. In this study, data on the Q‐angle, one of the check items, were extracted and investigated.　Ethics approval was obtained from the Ethics Committee of the National Institute of Sports Sciences (approval number: 28010). This study complied with the Strengthening of the Reporting of Observational Studies in Epidemiology guidelines. A research protocol was prepared, and written consent was obtained from all participants. The study was jointly designed by three Japanese physiotherapists with master's and doctoral degrees in sports and exercise physiotherapy.

### 2.2 Participants

The participants who underwent medical check‐ups at our facility between May 2001 and March 2021 were 11,616 top male and female athletes representing Japan who participated in international competitions such as the Olympics and Youth Olympic Games and Asian and World University Games. The target sports are 40 sports, including 33 sports at the Summer Olympics (Tokyo) and 7 sports at the Winter Olympics (Beijing). As mentioned earlier, medical check‐ups at our facility are mandatory before participating in international competitions, so all athletes who participated in international competitions during the period were eligible. For players who underwent medical check‐ups multiple times, only the data from the first visit was extracted and used for analysis. The exclusion criteria included missing data and difficulty in maintaining the knee joint in an extended position owing to pain or other factors.

### 2.3 Q‐Angle Measurement

The Q‐angle was measured using the method described in a previous study [1, 16], with the patient in a supine position and the quadriceps relaxed. Before measurements, the borders of the patella, tibial tubercle, and anterior superior iliac spine were identified by careful palpation, and the center of the patella and tibial tubercle were marked.　The angle between the line from the anterior superior iliac spine to the center of the patella and the line from the center of the patella to the center of the tibial tuberosity was measured for both lower limbs using a goniometer. For safety concerns, measurements were not performed in patients who could not conveniently maintain knee joint extension owing to pain or other factors. Measurements were carried out by qualified persons such as physical therapists and athletic trainers who had undergone sufficient training in advance. In addition, at the time of measurement, a recorder different from the measurer checked the measurement procedure.

### 2.4 Data Analysis

Previous studies defined a Q‐angle measurement of > 20° as a positive high Q‐angle [11]. Additionally, an absolute difference between the left and right Q‐angles of ≥ 3° was defined as a positive asymmetric Q‐angle [17]. Subsequently, the participants were classified according to the presence or absence of positivity based on each criterion, and the positivity rates of high and asymmetric Q‐angles were evaluated. Sex differences in the positivity rates of high and asymmetric Q‐angles were examined as follows age was entered as a covariate, and logistic regression analysis was performed, with the dependent variable being the presence or absence of a positive high or asymmetric Q‐angle, to calculate the odds ratio (OR) of the positivity rate in female compared with that in male. These statistical analyses were carried out using the methods of previous studies [18]. The results were analyzed using the statistical analysis software SPSS (version 29.0; IBM Corp.), and the significance level was set at 5% for two‐sided tests.

### 2.5 Patient and Public Involvement

The study participants were pre‐international competition athletes and therefore were not involved in the design, conduct, interpretation, or translation of the current study.

## 3 Results

### 3.1 Participant Demographic Characteristics

**Figure 1:**
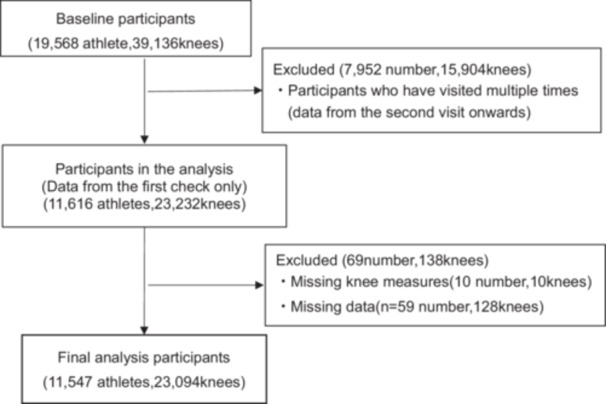
Flow chart study participants.

Data from 7952 participants (15,904 knees) who underwent medical assessments multiple times during the study period were excluded. Additionally, data of 138 knees of 69 individuals were missing or could not be assessed; therefore, they were excluded from the analysis (Figure 1, Table 1). Finally, data of 11,547 athletes (23,094 knees), from 6725 male athletes (13,450 knees) and 4822 female athletes (9644 knees) of 40 sports were analyzed (Figure 1, Table 1).

**Table 1:** List of participants' competitions.

Competition	Total (athlete)	Male (athlete)	Female (athlete)
Summer olympic games (33 sports)			
Archery	231	106	125
Athletics	1343	806	537
Badminton	212	101	111
Baseball/Softball	572	405	167
Basketball	661	316	345
Boxing	91	80	11
Canoeing	136	95	41
Cycling	317	240	77
Equestrian	58	43	15
Fencing	238	114	124
Football	1009	688	321
Golf	226	101	125
Gymnastics	364	121	243
Handball	237	131	106
Hockey	216	114	102
Judo	240	116	124
Karate	56	28	28
Modern pentathlon	48	33	15
Rowing	153	91	62
Rugby	593	496	97
Sailing	189	108	81
Shooting	139	62	77
Skateboarding	44	21	23
Sport climbing	46	25	21
Surfing	6	3	3
Swimming	645	326	319
Table tennis	170	89	81
Taekwondo	78	46	32
Tennis	123	57	66
Triathlon	111	49	62
Volleyball	576	276	300
Weightlifting	126	76	50
Wrestling	177	121	56
Winter Olympics (7 sports)			
Biathlon	54	31	23
Bobsleigh	77	46	31
Curling	116	46	70
Ice hockey	450	272	178
Luge	20	12	8
Skating	592	309	283
Skiing	807	525	282
Total (athletes)	11,547	6725	4822

*Note:* Extract only data from the first medical check‐ups.

### 3.2 Age and Q‐Angle Measurements of the Participants

The age of the participants was 20.8 (18.5–23.4) years overall, with male and female athletes aged 21.3 (19.0–24.1) years and 20.0 (18.0–22.2) years, respectively. The Q‐angles were 13.0° (10.0–15.0) overall, with 12.0° (10.0–14.5) and 15.0° (12.0–17.0) for male and female athletes, respectively (Table 2).

**Table 2:** Participant age and quadriceps angle measurement results.

	Total (*n* = 11,547)	Male (*n* = 6725)	Female (*n* = 4822)
Age (y)	20.8 (18.5–23.4)	21.3 (19.0–24.1)	20.0 (18.0–22.2)
Q‐angle (°)	13.0 (10.0–15.0)	12.0 (10.0–14.5)	15.0 (12.0–17.0)

*Note:* Values are presented as median (interquartile range).

### 3.3 Positivity Rate of High Q‐Angle and Sex Differences

A total of 1837 knees were positive for high Q‐angle, with a positivity rate of 8.0%, of which 565 were positive in male athletes (4.2% positivity rate) and 1272 were positive in female athletes (13.2% positivity rate). The positivity rate of high Q‐angles in female athletes was significantly higher than that in male athletes, with an OR of 3.47 (*p *< 0.05, 95% CI: 3.13–3.84) (Table 3).

**Table 3:** Positivity rates of high and asymmetric quadriceps angles and sex differences.

		Sex			95％ Confidence interval		
	Total	Male	Female	OR	Lower	Upper	*p* value
Presence or absence of high‐Q angle							
Positive (knee)	1837	565	1272	3.47	3.13	3.84	＜0.05
Negative (knee)	21,257	12,885	8372				
Positivity rate (％)	8.0	4.2	13.2				
Total (knee)	23,094	13,450	9644				
Presence or absence of asymmetry in the Q‐angle							
Positive (number)	2872	1528	1344	1.26	1.16	1.34	＜0.05
Negative (number)	8675	5197	3478				
Positivity rate (％)	24.9	22.7	27.9				
Total (number)	11,547	6725	4822				

### 3.4 Positivity Rate of Asymmetric Q‐Angle and Sex Differences

Overall, 2872 knees were positive for asymmetric Q‐angle, with a positivity rate of 24.9%. In male athletes, 1528 knees were positive, with a positivity rate of 22.7%, whereas in female athletes, 1344 knees were positive, with a positivity rate of 27.9%. The positivity rate of asymmetric Q‐angle was significantly higher in female athletes than in male athletes, with an OR of 1.26 (*p* < 0.05, 95% CI: 1.16–1.34).

## 4 Discussion

To our knowledge, this study is the first to analyze the characteristics of athletes' Q‐angles, focusing on the presence or absence of high Q‐angles and left‐right asymmetry, using a large‐scale survey of elite athletes representing Japan in various summer and winter sports for approximately 20 years. In past reports, surveys were conducted on only certain sports, and the sample size was limited, so it was unclear how many athletes potentially have Q‐angle malalignment. Therefore, the study results have high clinical significance. Additionally, from an academic perspective, we believe that this study may provide important insights and basic knowledge for future research into knee injuries in athletes.

The positivity rate of high Q‐angles was 8.0% overall, with a significantly higher rate in female athletes (13.2%) than in male athletes (4.2%); an OR of 3.47 indicated a sex difference. Horton and Hall [2] surveyed high Q‐angles in non‐athletes and reported no positive knees in male participants; however, the positivity rate was approximately 15% in female participants of that study. As sex differences have also been observed in previous studies, our findings support those of previous reports, and the results were in line with our hypothesis. Reportedly, quadriceps strength [2, 9, 11] is related to the resistance of the quadriceps to the lateral force of the patella. Additionally, a wider pelvis may place the reference point for the Q angle closer to the outside [19]. Studies of nonelite athletes have shown that a shorter femur may promote eversion of the leg and increase the Q angle [20]. The sex differences observed in this study are thought to be influenced by these differences in the musculoskeletal system between males and females. In addition, the positive rate in non‐female athletes in Horton and Hall [2] was 15%, while the positive rate in female elite athletes in this study was 13.2%. These results support the hypothesis that differences between elite athletes and nonathlete subjects, namely differences in the musculoskeletal system such as the quadriceps, may affect the Q angle. Elite athletes often routinely exercise their quadriceps during practice and training to improve their performance. They also stretch the muscles around the knee, which are overworked during sports activities and can lead to increased tightness and poor conditioning. Consequently, it is suggested that the daily training and conditioning of the quadriceps and other muscles that elite athletes undertake may alter the Q angle. Regarding asymmetric Q‐angles, Raveendranath et al. [21] conducted a survey of the asymmetry of Q‐angles in non‐athletes and reported that approximately 96% of male and female subjects had a difference of less than 3°. Therefore, in this study, a difference of ≥ 3° was defined as a positive asymmetric Q‐angle. Thus, 24.9% of the participants (22.7% male and 27.9% female) tested positive. This study revealed that the proportion of athletes with asymmetric Q‐angles was higher than that of non‐athletes [16]. This result supported the hypothesis. This is possibly influenced by the sports' characteristics. The elbow valgus angle of baseball players is increased on the throwing side compared with that on the non‐throwing side owing to the repeated valgus stress caused by pitching movements [22, 23]. It is presumed that the Q‐angle is also affected by attributes such as the dominant or nondominant foot, right or left stance, and right or left rotation during athletic movements, such as running and jumping, which are performed daily by athletes, and that repeated valgus or varus stress is asymmetrically applied to the knee joint. Consequently, the difference between the left and right sides of the Q‐angle possibly increases, leading to an asymmetric Q‐angle as an adaptive response in sports. Although we did not conduct a detailed analysis, we believe that sports such as fencing events and track events in athletics, which are the sports our participants are involved in, routinely require asymmetric performance and are therefore likely to have a high degree of asymmetry. In addition, the positivity rate of asymmetric Q‐angles was higher in female than in male athletes, with an OR of 1.3. It was suggested that various factors, such as morphological differences in the pelvic girdle and lower limbs [2, 15], body weight differences [15], and morphological differences in the quadriceps [6, 11], may have influenced the positivity rate of asymmetric Q‐angles as well as high‐angle. Considering the results of this study and that an asymmetric Q‐angle is a risk factor for knee injuries, similar to a high Q‐angle [6, 9, 10], the significance of monitoring the occurrence of knee injuries in sports that involve asymmetric body control, particularly in female athletes, is emphasized. Additionally, quadriceps morphology, which may be related to the Q‐angle, is a modifiable factor. Focusing on female athletes, isometric quadriceps activation exercises such as quadriceps femoris muscle setting and straight leg raising are likely to be useful in improving Q‐angle malalignment^24^. Interventions focusing on the vastus medialis oblique muscle act not only proximally but also medially and posteriorly on the patella, and may be useful as the primary medial stabilizer of the patella because its tension functions to resist increases in the Q angle.^24^

### 4.1 Limitations

This study has some limitations. First, it did not fully consider the influence of the height and weight, pelvic girdle, lower limb length, and quadriceps morphology related to the Q‐angle. Consequently, differences in the musculoskeletal system may have influenced the results. Particularly, it is believed that elite athletes have well‐developed quadriceps muscles. Hence, when taking this into consideration, it is possible that the results may differ from those reported in previous studies involving non‐athletes. In the future, by researching groups with various athletic levels, not only will results be obtained that are applicable to that group, but it will also be possible to conduct a detailed analysis of the factors related to the Q‐angle. Additionally, analyzing the extent to which quadriceps morphology influenced the results is likely to provide useful insight when researching methods for preventing knee injuries. Alternatively, it may be useful to incorporate biomechanical analysis to better understand the basis of Q‐angle variability. We believe that more reliable results can be obtained by utilizing the latest technologies, such as 3D motion analysis and MRI. Furthermore, this study surveyed elite athletes, and the findings cannot be generalized to nonelite athletes. Additionally, the cross‐sectional design of this study might have strongly reflected the results of athletes in sports with a large number of participants. Lastly, while we did not investigate the relationship between high and asymmetric Q‐angles, previous studies have reported that high and asymmetric Q angles are associated with knee injuries [3, 6, 9]. Therefore, it is possible that high and asymmetric Q‐angles are risk factors. Although﻿, since most previous studies are cross‐sectional surveys, we believe that to consider high and asymmetric Q angles as risk factors, it is necessary to conduct longitudinal studies in the future.

## 5 Perspective

This study investigated high Q‐angles and left–right asymmetry in elite athletes of various sports and found that the prevalence was higher in females. This study uniquely contributes to the field by providing comprehensive insights into the prevalence of high and asymmetric Q‐angles across a broad spectrum of sports and highlighting sex disparities, thereby filling a crucial gap in existing sports medicine literature. Future research should consider morphological factors and their relationship to knee injuries, examining the factors that contributed to the findings of this study and the risk of knee injuries. Additionally, clarifying the characteristics of the Q‐angle in each sport and comparing the Q angle between sports with different movement characteristics will facilitate the prediction of sports in which knee injuries occur frequently, and will also make it possible to understand what sport characteristics are related to increases and decreases in the Q‐angle. For example, it is useful to compare sports that primarily use one foot (e.g., soccer, tennis) with sports that primarily use both feet (e.g., weightlifting, cycling). It is also important to conduct sensitivity analyses, excluding sports with very few participants, to see if and how the results change. This would assess the robustness of our findings and determine whether certain sports have a disproportionate influence on the overall results, providing further insights. We believe that this knowledge will aid in considering injury prevention methods in the future. In addition, we believe that investigating the effect of age on the Q angle in elite athletes using a longitudinal study design may contribute to the prevention of knee injuries in veteran athletes. Simultaneously, Longitudinal studies following athletes over multiple seasons could provide invaluable insights into the temporal relationship between Q‐angle changes and knee injury development, offering a more definitive conclusion on causality. Furthermore, future research should explore interventions, such as strength training programs or corrective exercises, to assess their efficacy in modifying high and asymmetric Q‐angles and reducing injury risk among elite athletes, especially female athletes. A multidisciplinary approach involving collaboration among experts in biomechanics, physiotherapy, sports medicine, and athletic training will be crucial for translating these findings into effective injury prevention strategies.

## 6 Conclusion

To our knowledge, this is the first study to involve a large‐scale investigation of Q‐angles in elite athletes and to clarify the characteristics of high and asymmetric Q‐angles. This study provides comprehensive insights into the prevalence of high and asymmetric Q‐angles across a broad spectrum of sports and highlights sex disparities, thereby filling a crucial gap in existing sports medicine literature. Compared with male athletes, female athletes were more likely to have high and asymmetric Q‐angles. In addition, consistent with previous studies on non‐athletes, this study suggested that athletes may have a higher proportion of asymmetric Q‐angles than non‐athletes do. Therefore, given the significantly higher prevalence of high and asymmetric Q‐angles in female athletes, our findings underscore the need for sex‐specific approaches in injury prevention programs. These should be integrated into sports health management practices to identify athletes at higher risk of knee injuries and implement targeted preventive interventions. While our study provides valuable insights, it is important to note that the cross‐sectional design limits our ability to infer causality between Q‐angle characteristics and injury occurrence. Additionally, the lack of detailed information on the athletes' training regimes and injury history precludes a more nuanced understanding of the interplay between Q‐angle dynamics and knee injury risks.

## Author Contributions

**Kohei Hikawa:** conceptualization, data curation, formal analysis, validation, and writing – original draft. **Reia Shimizu:** conceptualization, data curation, validation, writing – review and editing. **Saeko Takahashi:** conceptualization, funding acquisition, and supervision.

## Ethics Statement

The study was approved by the Ethics Committee of the National Institute of Sports Sciences, approval number: 28010.

## Consent

Informed consent was obtained, and written consent was obtained.

## Conflicts of Interest

The authors declare no conflicts of interest.

## Transparency Statement

The lead authors, Kohei Hikawa and Saeko Takahashi, affirm that this manuscript is an honest, accurate, and transparent account of the study being reported; that no important aspects of the study have been omitted; and that any discrepancies from the study as planned (and, if relevant, registered) have been explained.

## Data Availability

All data relevant to the study are included in the article or are available as supplemental files. None of the data sets included in this study were identifiable.
